# Acylation modification mediated post-translational modifications learning signature reveals ZDHHC18 promotes progression of lung adenocarcinoma by attenuating immunocyte activation

**DOI:** 10.3389/fimmu.2026.1802631

**Published:** 2026-04-07

**Authors:** Qifan Xiao, Jianlong Bu, Mengfeng Liu, Lei Shi, Changfa Qu

**Affiliations:** 1 Department of Thoracic Surgery, Harbin Medical University Cancer Hospital, Harbin, China; 2 Department of Thoracic Surgery, Beidahuang Industry Group General Hospital, Harbin, China

**Keywords:** acylation modification, immunotherapy, lung adenocarcinoma, post-translational modifications (PMTs), ZDHHC18

## Abstract

**Background:**

Although the acylation modification (AM) significantly influences the development of lung adenocarcinoma (LUAD), the specific mechanisms of acylation modification in this context have not been widely researched. This investigation sought to discover novel therapeutic avenues related to acylation modification for the precision treatment of LUAD.

**Methods:**

Multiomics consensus clustering was generated for 12 types of acylation modifications, including crotonylation, lactylation, succinylation, benzoylation, acetylation, malonylation, glutarylation, 2-hydroxyisobutyrylation, β-hydroxybutyrylation, palmitoylation, myristoylation, and prenylation. Then, we established a learning signature referred to as AM.score using machine learning from 1,720 LUAD patients, and was validated across 8 cohorts and in-house cohort. We conducted a thorough evaluation involving 9 distinct immunotherapy cohorts to assess the effectiveness of the AM.score in predicting responses to immunotherapy treatments. Finally, the function of ZDHHC18 in LUAD was conducted both *in vivo* and *in vitro*.

**Results:**

The developed AM.score demonstrated remarkable predictive capabilities in forecasting outcomes for LUAD, and outperformed currently utilized prognostic indicators specific to LUAD. Furthermore, the predictive power of AM.score was not confined to LUAD alone; it was validated across various categories of malignancies, showcasing its broad applicability in evaluating responses to immunotherapy. From a biological standpoint, the analysis revealed significant correlations between AM.score and various immunological parameters. Notably, higher levels of AM.score were associated with induced immune responses, which in turn were linked to characteristics commonly associated with immunologically hot tumors-those that can elicit a robust immune response. Comprehensive scRNA-seq and spatial transcriptomics analyses demonstrated that elevated AM.score was associated with increased cell malignancy. Then, ZDHHC18 has been recognized as an essential molecular element in the framework, and higher expression levels of ZDHHC18 was associated with poorer clinical outcomes in LUAD. Mechanistically, knockout ZDHHC18 simultaneously enhance strong immune responses that hinder LUAD progression.

**Conclusion:**

AM.score functions as a crucial predictive biomarker, providing valuable insights into both prognosis and the suitability of immunotherapy for individual patients. Furthermore, the focus on targeting ZDHHC18 presents a promising avenue for improving the effectiveness of immunotherapy.

## 1 Introduction

Lung adenocarcinoma (LUAD) represents the predominant histological subtype of lung cancer, and its high fatality rate is primarily attributable to multiple factors, including the lack of pronounced early symptoms and the development of resistance to existing therapeutic approaches (1). Despite advances in treatment modalities such as surgery, chemotherapy, radiotherapy and targeted therapy, overall survival rates for LUAD remain low, with particularly poor prognoses for patients at advanced stages (2). In recent years, the advent of ICI therapy has brought renewed hope for the treatment of lung adenocarcinoma. However, a significant proportion of LUAD remain unresponsive or develop resistance to immunotherapy. Some patients develop resistance shortly after treatment initiation, leading to disease progression and recurrence. Furthermore, substantial variations in treatment response and clinical outcomes persist among patients receiving identical immunotherapy regimens (3, 4). Therefore, identifying more precise molecular biomarkers for early diagnosis, prognostic assessment, and personalized treatment represents an urgent requirement for improving clinical outcomes in patients with LUAD.

Protein modifications have been demonstrated to be closely associated with the onset of LUAD. Post-translational modifications of proteins encompass over 400 forms, including acylation, methylation, phosphorylation, and ubiquitination (5). Among these, acylation modification (AM) is the most prevalent and significant form of protein modification, occurring in 80% to 90% of eukaryotic cells (5). Research indicates that impaired histone acetylation modifications are associated with the onset of LUAD. Research indicates that elevated expression of HDAC1 and HDAC3 correlates with poor prognosis in LUAD, whereas reduced expression of HDAC5 and HDAC6 is associated with unfavorable outcomes in LUAD (6). Succinylation can impact the functionality of T-cells by modifying ZAP70, a critical protein involved in T-cell signaling pathways (7). Additionally, lactylation has been found to be associated with immune evasion mechanisms in cancer, demonstrating how cancer cells can evade detection and destruction by the immune system (8, 9). Furthermore, the regulation of immune checkpoint molecules by AM is pivotal in determining the effectiveness of immunotherapy strategies (10). Emerging evidence suggests that a variety of AMs are involved in the complex regulation of immune functions across different types of cancers, including LUAD. These modifications not only affect tumor immune evasion but also contribute to resistance against therapeutic interventions, highlighting the intricate interplay between AMs and cancer biology.

Although considerable advancements have been made in this area, fully grasping the link between AM and LUAD remains difficult. The complex relationship between AM and the alterations in clinical features related to LUAD requires more in-depth research to elucidate these connections. In order to tackle the existing gaps in our knowledge concerning potential biomarkers for LUAD, we adopted a comprehensive and integrative methodology. Our primary objective throughout this research was to identify specific biomarkers that could improve both the prognostic process and the overall understanding of LUAD. To achieve this, we harnessed the power of advanced machine learning algorithms, which allowed us to create a novel measurement tool referred to as AM.score. This innovative metric is designed to predict the efficacy of treatments in patients suffering from LUAD, thereby offering new insights into patient management and care. Our research has convincingly shown that AM.score is highly effective in forecasting clinical outcomes for LUAD, potentially aiding in the personalization of treatment strategies based on predicted outcomes. Furthermore, through a comprehensive analysis, we have pinpointed ZDHHC18 could serve as promising targets for LUAD. By identifying ZDHHC18’s involvement, our findings highlight its critical importance in the intricate mechanisms that underlie LUAD, opening new avenues for further research and potential therapeutic interventions.

## 2 Materials and methods

### 2.1 Data acquisition

Initially, we acquired eight datasets containing prognostic information, specifically TCGA-LUAD, GSE41271 (11), GSE37745 (12), GSE42127 (13), GSE50081 (14), GSE72094 (15), GSE13213 (16), and GSE31210 (17). This comprehensive collection of datasets will serve as a valuable resource for our research, enabling a more in-depth analysis of the relevant biological questions we aim to address. Additionally, to address and mitigate any batch effects that may arise between platform, removeBatchEffect in R software will be employed (**Supplementary Figures 1A, B**) (18). This function is instrumental in standardizing the data by adjusting for systematic variations that could skew the results, thereby enhancing the reliability and validity of the findings. Additionally, we compiled a comprehensive collection of nine immunotherapy cohorts. This collection comprises five cohorts specifically dedicated to melanoma immunotherapy, which includes data from studies such as Van Allen (19), Nathanson (20), GSE35640 (21), GSE78220 (22), and GSE91061 (23). Moreover, we have also integrated a cohort that focuses on metastatic urothelial cancer, known as the IMvigor210 cohort (24). To further enrich our analysis, we included a lung cancer immunotherapy cohort, which encompasses data from the GSE126044 (25), GSE135222 (26), and GSE136961 (27) studies. We retrieved acylation modification from published literature (28) (**Supplementary Table 1**).

### 2.2 Multiomics consensus clustering

We employed the “getElites” function from the MOVICS package to identify and evaluate gene features effectively. This tool facilitated a comprehensive analysis of the genetic data, allowing us to focus on the most significant attributes relevant to our study. By leveraging this advanced functionality, we aimed to enhance our understanding of the molecular underpinnings of cancer through a more refined selection of gene features (29).

### 2.3 Enrichment analysis

A comprehensive analysis of differential expression profiling was systematically carried out among the defined clusters of AM using the limma package. The criteria for significance were set at an FDR of less than 0.05 and |log2FC| greater than 2 (30). Subsequently, to further explore the biological implications of the identified gene expressions, GO and KEGG enrichment analyses were conducted. For these analyses, gene sets were carefully filtered based on specific parameters, including a size range of 5 to 5000 genes and a significance threshold of *p* less than 0.05.

### 2.4 WGCNA

First, since network module analysis is susceptible to outliers, it is essential to remove outliers before constructing network modules to ensure the accuracy of results. Second, the pickSoftThreshold function was first used to calculate weight values and select an appropriate soft threshold. Subsequently, after determining the soft threshold, the formula dissTom = l - TOM was applied to eliminate errors caused by background noise and spurious associations. Finally, dynamic cutting is applied to segment the clusters generated by the cluster function (31).

### 2.5 Construction of AM.score

Genes satisfying the assumption of proportional hazard (P > 0.05) and VIF < 2 were selected to reconstruct the model. The TCGA-LUAD dataset played a crucial role in forming the basis of the risk model developed in this study. This foundational dataset provided essential insights and data that underpinned the model’s construction. In addition to this primary dataset, several other datasets were utilized to validate the model’s findings. Specifically, the GSE41271, GSE37745, GSE42127, GSE50081, GSE72094, GSE13213, and GSE31210 datasets were instrumental in corroborating the results. This multi-dataset validation approach enhances the robustness and reliability of the findings, ensuring that the constructed risk model is well-supported by a comprehensive analysis of diverse data sources.

### 2.6 Single cell RNA and spatial transcriptomics data processing and analysis

We utilized scRNA data sourced from the GSE189357 (32) dataset and ST data sourced from the GSE179572 dataset (33). The data processing and analysis were in **Supplementary Material**.

### 2.7 Immunohistochemical staining and analysis

A total of 50 LUAD patients who underwent primary surgical resection were enrolled in this study. The study protocol was approved by the Ethics Committee of the Harbin Medical University Cancer Hospital (Approval No. 2025-036). None of the patients had received any preoperative therapy prior to surgical intervention. IHC staining and analysis were conducted on 3 μm sections obtained from 50 patients, which were processed by Biossci Biotechnology Co. Ltd. Following a series of steps including deparaffinization, antigen unmasking, and blocking, the slides were incubated overnight at 4 °C with a rabbit-derived anti-CD8 antibody (ab217344, Abcam) at a dilution of 1:16000 and rabbit-derived anti-IFIT1 antibody (83423-1-RR, Proteintech) at a dilution of 1:200. Subsequently, the sections underwent DAB staining and were counterstained using hematoxylin.

### 2.8 RNA extraction and quantitative real-time PCR

Total RNA from LUAD was isolated using the Quick RNA Extraction Kit (AG21023, Accurate Biotech, Changsha, China), following the manufacturer’s protocol. RNA concentrations were assessed using a NanoDrop spectrophotometer (ThermoFisher). Complementary DNA (cDNA) synthesis was carried out using the Evo M-MLV RT Premix (AG11706, Accurate Biotechnology, Hunan, China). Quantitative real-time PCR (qRT-PCR) was performed on the CFX96 Touch Real-Time PCR System (Bio-Rad, USA) utilizing the SYBR^®^ Green Premix Pro Taq HS qPCR Kit (AG11701, Accurate Biotechnology, Hunan, China). To ensure accurate quantification, GAPDH served as an endogenous reference for normalizing RNA expression levels. The relevant RNA sequences are listed in **Supplementary Table 2**.

### 2.9 Cell lines

A549 and H1299 cells were purchased from the Cell Bank of the Chinese Academy of Sciences (Shanghai, China). All cell lines were periodically authenticated via STR profiling (Procell, China). A549 and H1299 cells were cultured in DMEM (Wisent) with 10% FBS (Wisent) and 1% penicillin/streptomycin (Wisent). An incubator with 5% CO2 was used to keep all of the cells at 37 °C. The relevant RNA sequences are listed in **Supplementary Table 2**.

### 2.10 Mouse model

Experimental 8-week-old female C57BL/6 mice were obtained from Gempharmatech, located in Jiangsu, China. A study was conducted using C57BL/6 mice, which were randomly divided into three distinct experimental groups: normal mice (n=10), siNC (n=10), and siZDHHC18 (n=10). The xenograft model was established by subcutaneous administration of siNC, siZDHHC18 LLC cells (2×10^5^ cells/100 µL PBS). Therapeutic intervention commenced on tumors reaching 100 mm^3^. The suspension is composed of pre-cooled PBS and Matrigel (Corning; Cat#354234) at a 1:1 ratio. Using an insulin syringe, inject 100 μL of the cell suspension subcutaneously into each animal. Mice are randomly assigned and treated according to the treatment protocol. The development of subcutaneous xenograft tumors is observed daily, and the body weight of tumor-bearing animals is dynamically monitored every 2 days. Tumor size is recorded using a caliper to calculate tumor volume for subsequent analysis. At the end of the experiment, tumor size and weight, as well as mouse body weight, are recorded as experimental data. The isolated tumors and organ sections are used for subsequent flow cytometry analysis.

### 2.11 Flow cytometry

To detect immune markers on the cell membrane, collected cell samples were washed with PBS and stained with the required flow cytometry antibodies and their isotype control antibodies diluted in pre-cooled PBS. After mixing thoroughly, the samples were incubated at 4 °C in the dark for 30 minutes, followed by washing and resuspension in PBS for detection. The immune cells were then incubated with specific antibodies as detailed in **Supplementary Table 3**. Flow cytometry data were collected using the Cytek Aurora or Cytoflex flow cytometer. Flow cytometer parameters were adjusted based on forward and side scatter, side scatter height vs. side scatter width, and forward scatter area vs. forward scatter height to ensure appropriate flow cytometry voltage. The collected data were analyzed using FlowJo software (version 10.8).

### 2.12 Statistical analysis

Continuous data were expressed as the mean value along with the standard deviation, represented as mean ± standard deviation (mean ± SD). To evaluate the differences between groups, two-tailed unpaired Student’s t-tests were utilized. All statistical analyses were conducted using R software, specifically version 4.5.1. For the purposes of this study, statistical significance was determined by a p-value of less than 0.05.

## 3 Results

### 3.1 Identification of AM related subtypes

**Figure 1:**
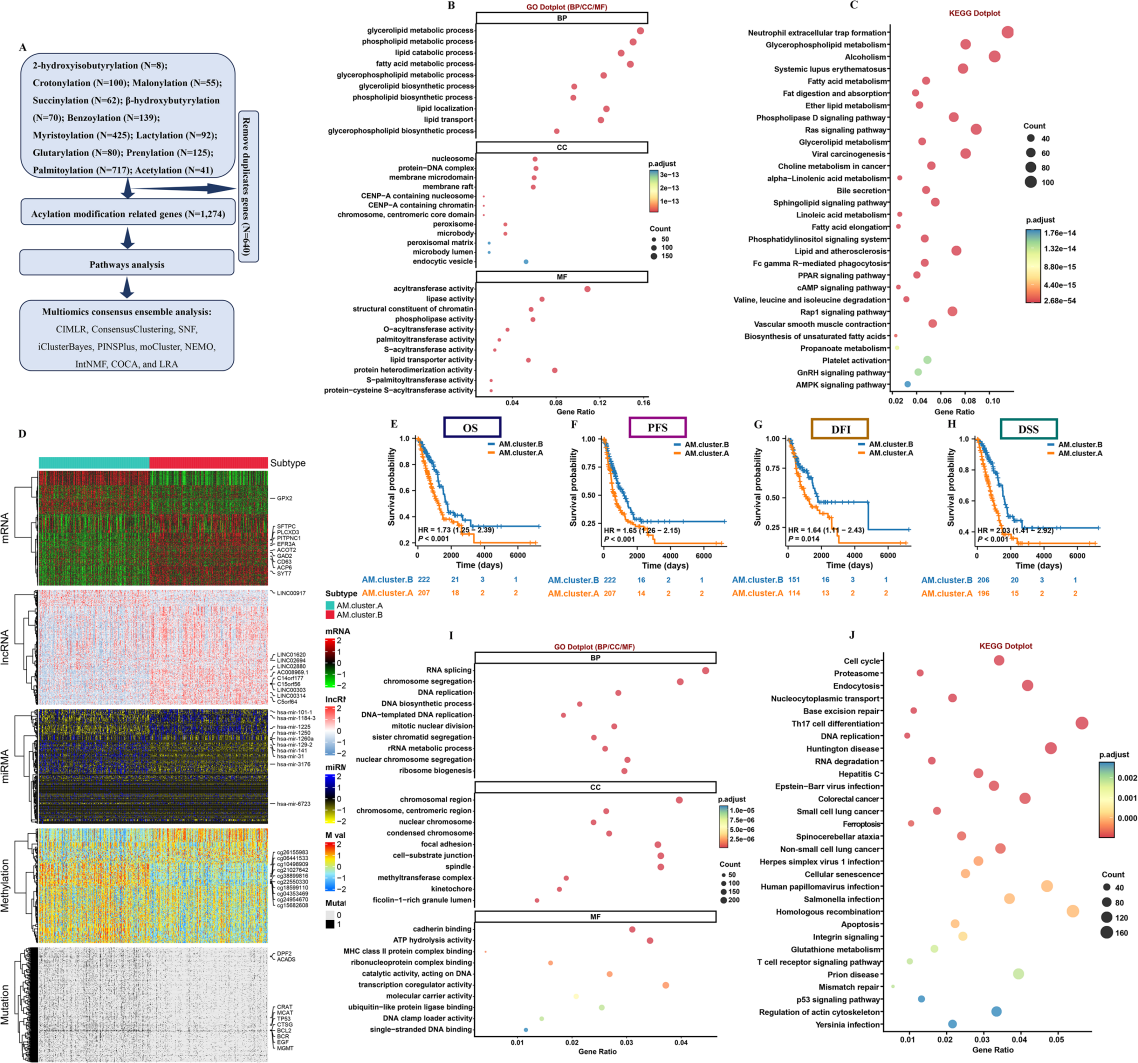
Identification of molecular subtypes of LUAD. **(A)** The process of acylation modification related genes search. **(B)** GO and **(C)** KEGG enrichment analysis on acylation modification related genes. **(D)** Comprehensive heatmap of consensus ensemble subtypes. Different OS **(E)**, PFS **(F)**, DFI **(G)**, DSS **(H)** outcomes among the two subtypes. GO **(I)** and KEGG **(J)** enrichment analysis on the DEGs between the 2 clusters.

Firstly, 1,274 genes established as core regulators of AM, were designated as AM related genes for subsequent molecular characterization (**Figure 1A**). Following this, GO and KEGG analyses were performed. These analyses highlighted that a substantial number of the genes investigated are integral to AM pathways, in addition to being associated with pathways related to LUAD (**Figures 1B, C**). Then, multiomics consensus clustering was developed by employing 10 clustering techniques. The results demonstrated that the clustering yielded the most optimal outcomes (K = 2) (**Figures 1D, E**, **Supplementary Figures 2A, B**). Additional analysis of survival results revealed that individuals categorized under AM.cluster.A exhibited notably worse OS, DSS, PFS, DFI when compared to those identified as belonging to AM.cluster.B (**Figures 1E–H**). Next, 6,226 DEGs were established (**Supplementary Table 4**). DEGs were largely concentrated in immune pathways associated with LUAD (**Figures 1I, J**). Their involvement in these specific pathways underscores their potential importance in the biological mechanisms underlying LUAD.

### 3.2 WGCNA analysis

**Figure 2:**
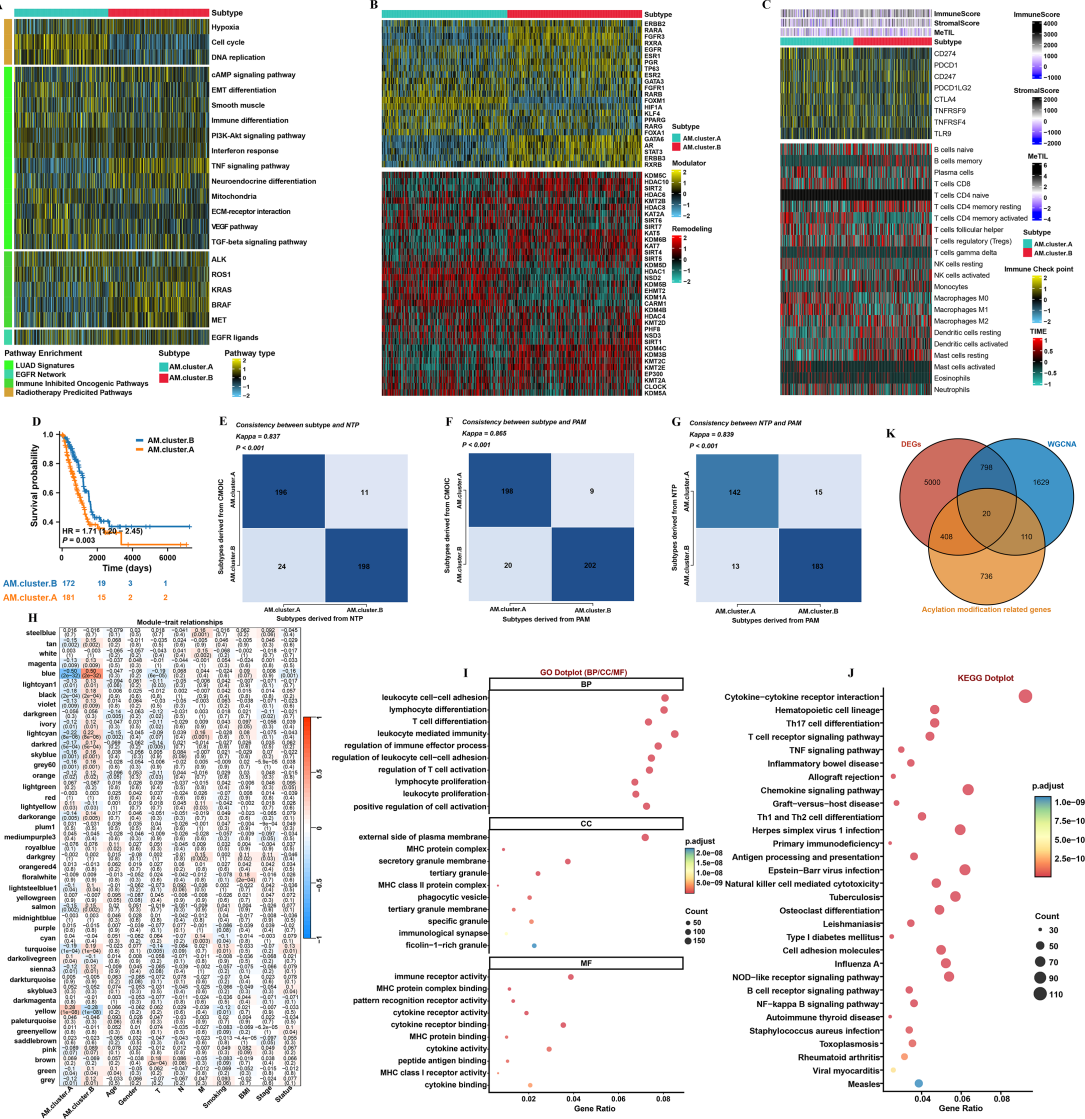
WGCNA analysis. **(A)** The enrichment of two subtypes corresponding to LUAD associated signatures. **(B)** Activity profiles of 23 transcription factors (top) and possible regulators linked to chromatin remodeling (bottom) for the two subtypes. **(C)** Immune characteristics within the TCGA-LUAD cohort. **(D)** Survival analysis of two subtypes. **(E)**The consistency of subtypes with NTP. **(F)** The consistency of subtypes with PAM. **(G)** The consistency of NTP with PAM. **(H)** Correlation analysis between module eigengenes and clinical traits. **(I)** GO and **(J)** KEGG enrichment analysis on the key module genes. **(K)** Venn diagram illustrating the identification of 20 overlapping hub genes.

Currently, the classification of various molecular subtypes of LUAD primarily hinges on the levels of molecular expression, which may correlate with distinct biological functions. Our analysis revealed noteworthy variations in the responsiveness to specific treatment options among the identified subtypes. For instance, AM.cluster.B exhibited significant enrichment in pathways that are associated with immune-inhibitory oncogenic processes. Conversely, AM.cluster.A appeared to have a greater likelihood of benefiting from therapeutic interventions such as radiotherapy and targeted therapy (**Figures 2A-C**). The nearest template prediction (NTP) method serves as a powerful approach for categorizing samples within AMs. This systematic classification allows for the identification of specific subtypes that exhibit varying prognostic implications. Among these subtypes, AM.cluster.A, which was characterized using data from the TCGA-LUAD dataset, demonstrated the least favorable prognosis when compared to the other subtypes examined in the study (**Figure 2D**). Moreover, the analysis further investigated the consistency of the identified AM subtypes. This consistency was evaluated using both the NTP methodology and the partitioning around medoids (PAM) algorithms (**Figures 2E-G**).

Then, the findings of our analysis revealed that a β=4 produced R²=0.85 (**Supplementary Figure 3A**). After conducting this analysis, a dendrogram was constructed to visually represent the relationships among the different gene modules (**Supplementary Figure 3B**). This dendrogram was developed by carefully evaluating the variations that exist between these modules, providing insight into their interconnectedness. As a result of this thorough evaluation, researchers were able to identify a total of 45 unique gene modules. Each of these modules encapsulates a specific set of characteristics, highlighting their functional similarities and furthering our understanding of the genetic landscape. The analysis results indicated a considerable negative relationship between the blue module and AM.cluster.A (**Figure 2H**). Furthermore, enrichment analysis underscored the significant enrichment of the key module genes in biological processes that are associated with immune response (**Figures 2I, J**). Then, we conducted a meticulous selection process to identify a set of 20 overlapping genes that were present among the DEGs, key module genes and AM (**Figure 2K**). Moving forward, our upcoming research initiatives will center on these particular genes, with the goal of enhancing our comprehension of their functions and impacts on LUAD.

### 3.3 Construction of AM.score

**Figure 3:**
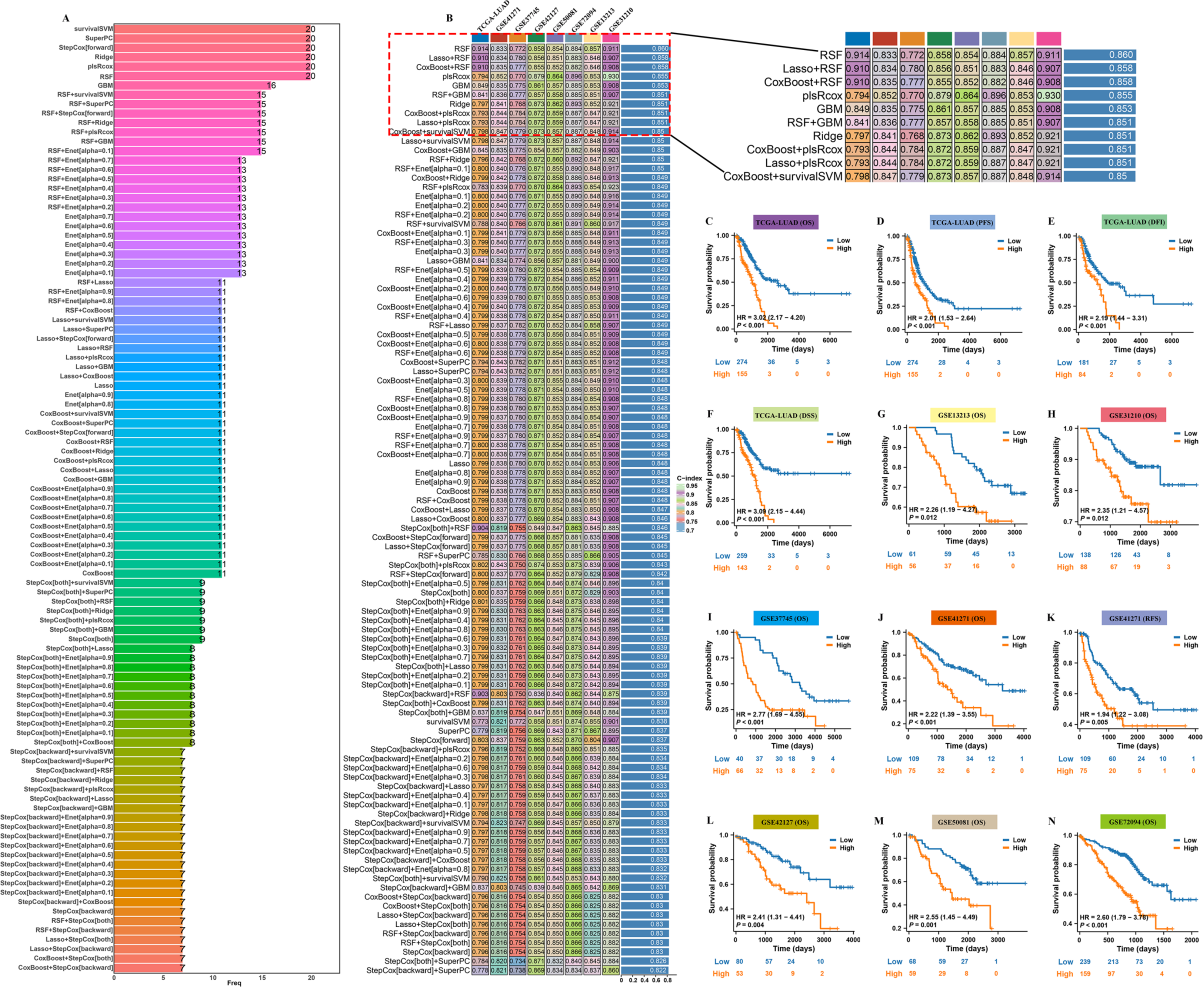
Construction of AM.score. **(A)** 20 most valuable genes based on the 101 combinations of machine learning algorithms. **(B)** A total of 101 combinations of machine learning algorithms for the AM.score via a 10-fold cross-validation framework. **(C-N)** Survival analysis of high and low AM.score groups.

Genes satisfying the assumption of proportional hazard (P > 0.05) and variance inflation factor (VIF) < 2 were selected to reconstruct the model. Subsequently, we checked for violations of the proportional hazard assumptions (all P >0.05; **Supplementary Table 5**). In addition, we found that the multicollinearity assumption was not violated, with all VIF < 2 (**Supplementary Table 6**). Then, the analysis revealed that the primary model that emerged was RSF, which is noteworthy for its impressive performance metrics. This hybrid model demonstrated exceptional capability, achieving an impressive average C index of 0.860. Such results highlight the effectiveness of AM.score in accurately distinguishing between relevant categories within the data (**Figures 3A, B**). Then, we divided the participants into high/low AM.score groups based on the median value of AM.score. Additional analysis of survival results revealed that individuals categorized under high AM.score exhibited notably worse survival when compared to those identified as belonging to low AM.score in the TCGA-LUAD, GSE41271, GSE37745, GSE42127, GSE50081, GSE72094, GSE13213, and GSE31210 datasets (**Figures 3C-N**). The predictive ability of AM.score for patient survival was also assessed using ROC curves, with AUC values exceeding 0.7 at one-, three-, and five-year intervals in TCGA-LUAD, GSE41271, GSE37745, GSE42127, GSE50081, GSE72094, GSE13213, and GSE31210 datasets, suggesting robust predictive capability (**Supplementary Figures 4A-H**). This particular feature elevates the model’s status as an essential asset in the field of risk assessment. It underscores the model’s critical role in guiding decision-making processes concerning risk management, demonstrating its significance in enhancing the effectiveness of risk-related strategies.

### 3.4 Evaluation of AM.score

**Figure 4:**
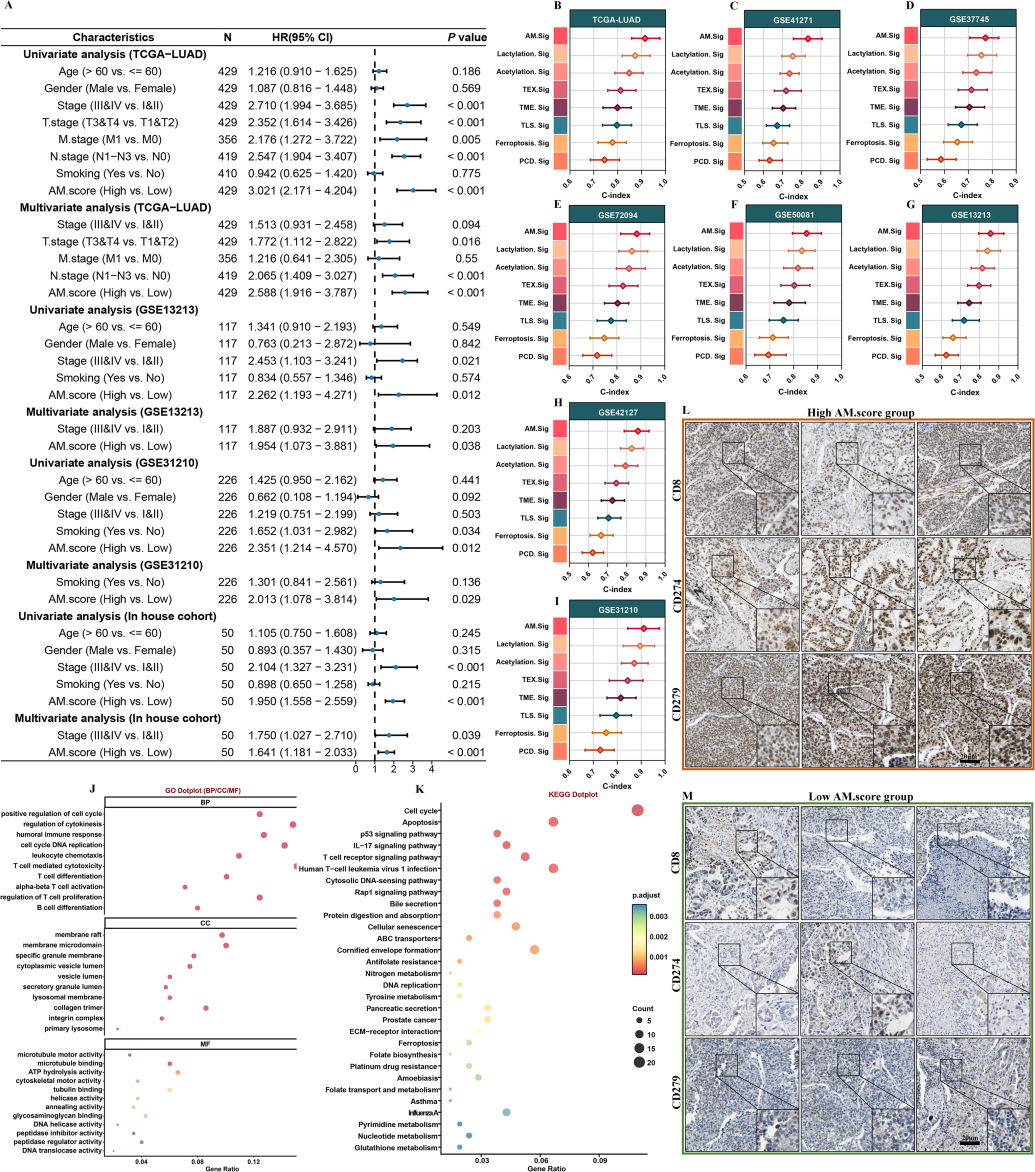
Evaluation of AM.score. **(A)** Univariate and multivariate Cox regression analyses of AM.score and clinicopathological characteristics. **(B-I)** Comparative analysis with 7 published signatures. **(I)** GO and **(J)** KEGG enrichment analysis on high and low AM.score groups. **(L, M)** Representative IHC staining images of CD8, CD274, and CD279 in high/low AM.score groups.

Subsequently, multivariate Cox regression indicates that the AM.score maintains its considerable significance even when accounting for additional clinical parameters that are known to impact patient outcomes (**Figure 4A**). To gain deeper insights into its effectiveness, we undertook a comparative analysis that involved juxtaposing it with a set of eight previously validated signatures targeting LUAD. Remarkably, the AM.score stood out by consistently exhibiting superior performance when measured against all other signatures that were included in our analysis across eight separate datasets. Additionally, it secured the highest C-index among all evaluated signatures, which emphasizes its potential role as a reliable and powerful predictive tool within this specific domain (**Figures 4B-I**, **Supplementary Table 7**). Next, upon identifying DEGs across varying AM.score, a comprehensive analysis revealed a total of 687 DEGs that were common between the two groups. Subsequent investigations indicated that these DEGs were primarily concentrated in a range of immune pathways associated with LUAD (**Figures 4J, K**). Additionally, the results of the immunohistochemical analyses provided further validation for these findings. The analyses revealed that the group characterized by a high AM.score exhibited significantly elevated levels of CD8A, PD-1, and PD-L1(**Figures 4L, M**). These findings underscoring high AM.score group are probably undergoing heightened immune activation.

### 3.5 Immunotherapy to AM.score

**Figure 5:**
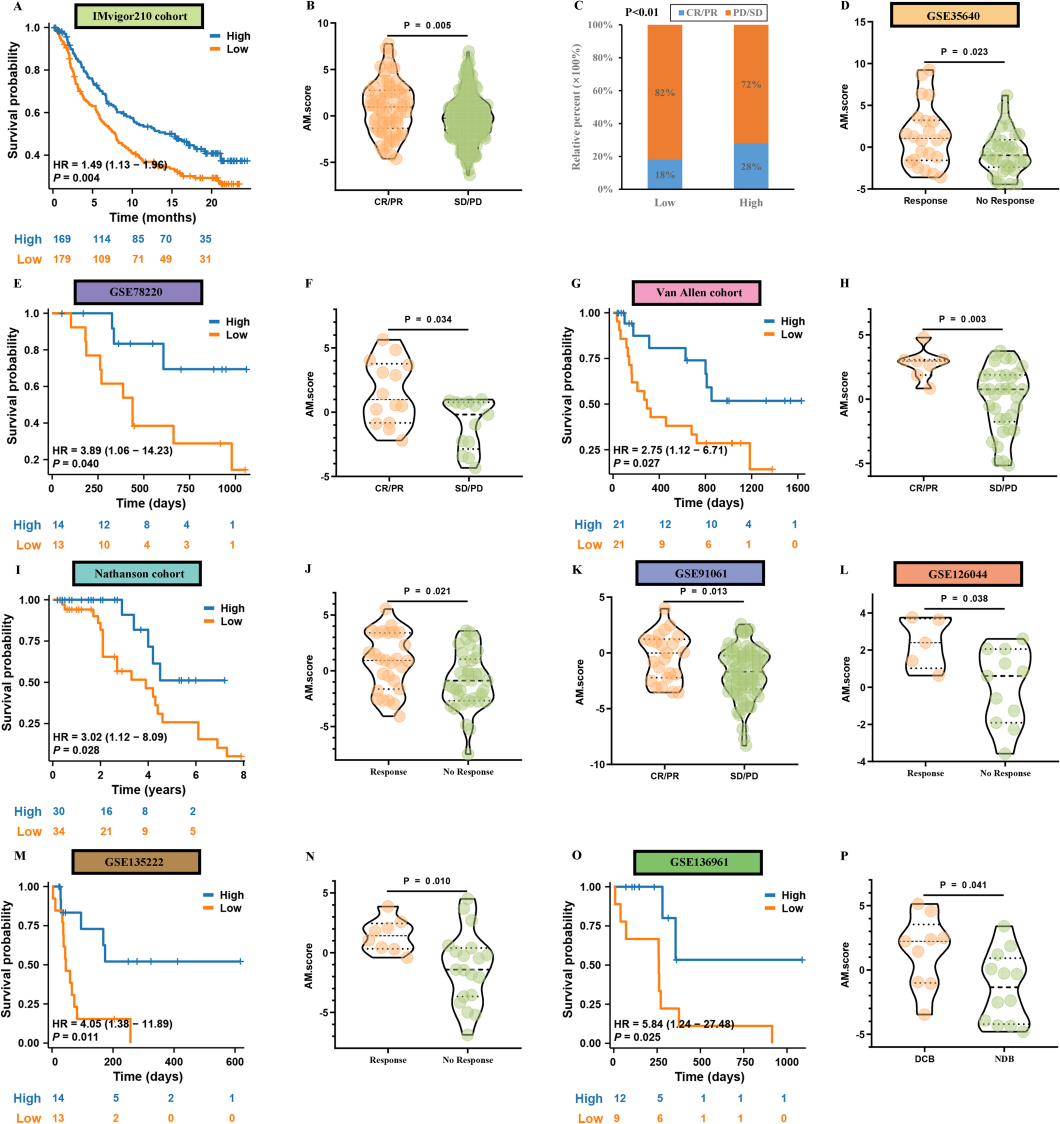
Immunotherapy to AM.score. **(K-M)** analysis of OS difference between the high and low AM.score in the IMvigor dataset **(A)**, GSE78220 **(E)**, Van Allen dataset **(G)**, Nathanson dataset **(I)**, GSE135222 **(M)**, GSE136961 **(O)**. Box plot displaying the AM.score in patients with different immunotherapy responses in the IMvigor dataset **(B, C)**, GSE35640 **(D)**, GSE78220 **(F)**, Van Allen dataset **(H)**, Nathanson dataset **(J)**, GSE91061 **(K)**, GSE126044 **(L)**, GSE135222 **(N)**, GSE136961 **(P)**.

Then, utilizing the 7 algorithms, our study revealed significant disparities in immune metrics when comparing two distinct groups defined by their AM.score. Significantly, the group showing a high AM.score revealed a pronounced rise in the levels of immune cell infiltration (**Supplementary Figure 5A**). In addition to the differences in immune cell infiltration, the levels of immune modulators were also found to be significantly greater in the high AM.score group than in low high AM.score group (**Supplementary Figure 5B**), which was consistent with the “immunity tidal model theory” that high expression of both costimulatory and coinhibitory immune checkpoints caused an immunosuppressive phenotype in tumors. Next, individuals who exhibited a low AM.score showed significantly poorer OS rates when compared to those with a high AM.score (**Figures 5A, E, G, I, M, O**). Furthermore, it was observed that the AM.score for the group categorized as having SD/PD was notably lower than that of the group recognized for their CR/PR (**Figures 5B-D, F, H, J-L, N, P**). This suggests that the level of the AM.score could be indicative of treatment efficacy, with higher scores associated with better therapeutic responses.

### 3.6 scRNA-seq data analysis AM.score

**Figure 6:**
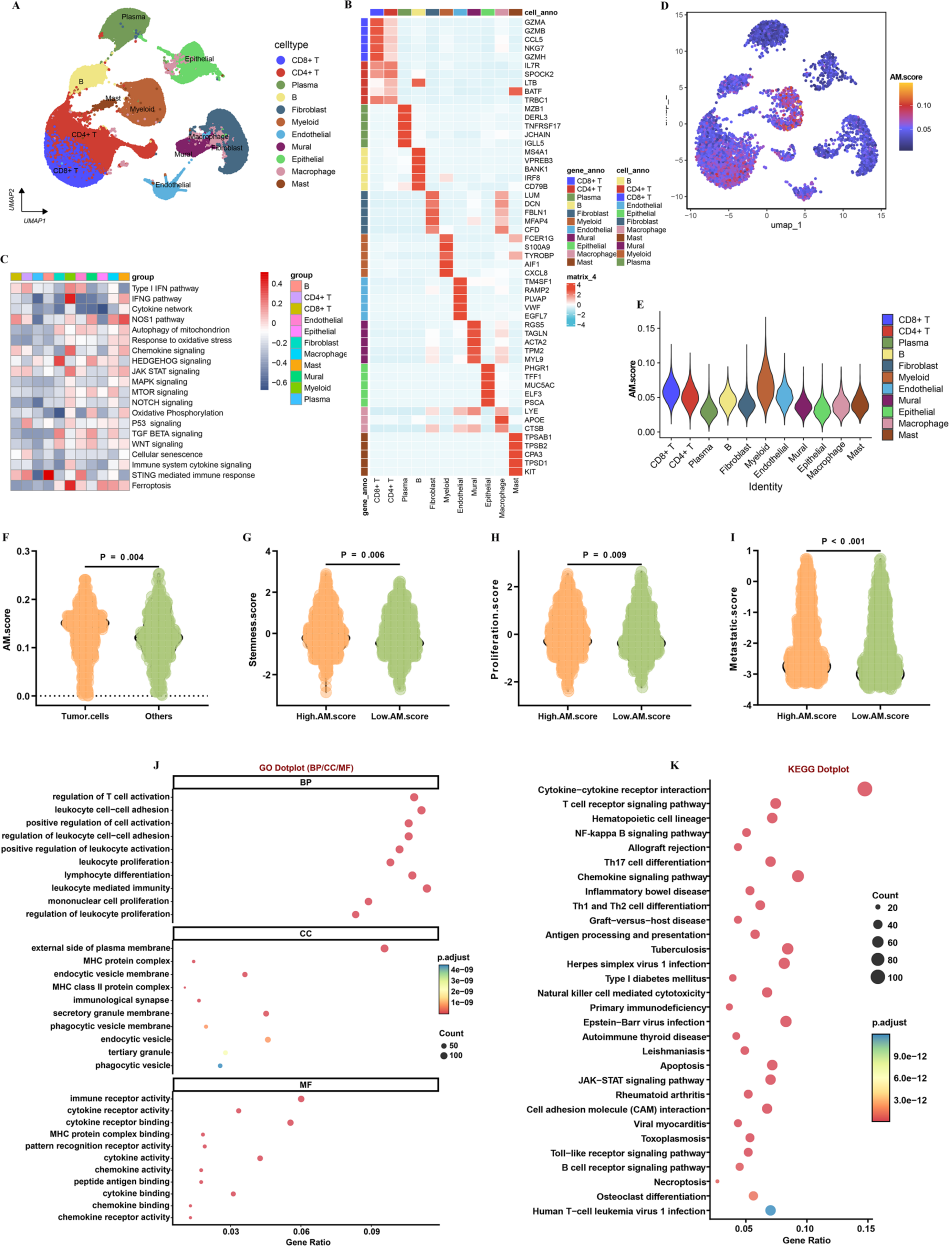
scRNA-seq data analysis AM.score. **(A)** 11 cell types were identified by performing UMAP. **(B)** Top 5 marker genes in each cell type. **(C)** Correlation of each cell type and main pathways. **(D, E)** The relative abundance of AM.score in cell types. **(F)** Illustrating the differences in AM.score between tumor cells and other cells. Violin plots displaying the expression scores of stemness **(G)**, proliferation **(H)**, and metastatic **(I)** in tumor cells from the high/Low AM.score. **(J)** GO and **(K)** KEGG analysis of DEGs between high/Low AM.score.

Based on the GSE189357 LUAD single-cell transcriptomics dataset, we annotated them into 11 cell types (**Figure 6A**). Furthermore, the analysis revealed specific cell types that are associated with distinct biological pathways (**Figure 6B**). The five most significant marker genes were highlighted, offering valuable insights that can inform future functional studies (**Figure 6C**). Then, we calculated the AM.score of each cell and found that myeloid and CD8+T cell had the highest scores (**Figures 6D, E**). The findings indicate that tumor cells exhibiting higher AM.scores demonstrate a markedly increased expression of various markers that are linked to stemness, proliferation, and metastasis. This correlation implies that tumors with elevated AM.scores may be inherently more aggressive and possess a more malignant phenotype. Consequently, this points to a potential association between high AM.scores and an enhanced degree of tumor malignancy, suggesting that AM.scores could serve as an important indicator in evaluating tumor behavior (**Figures 6F-I**). Following this, GO and KEGG indicated that DEGs identified between the high and low AM score groups are primarily involved in crucial immune-related pathways (**Figures 6J, K**).

### 3.7 ST data analysis AM.score

**Figure 7:**
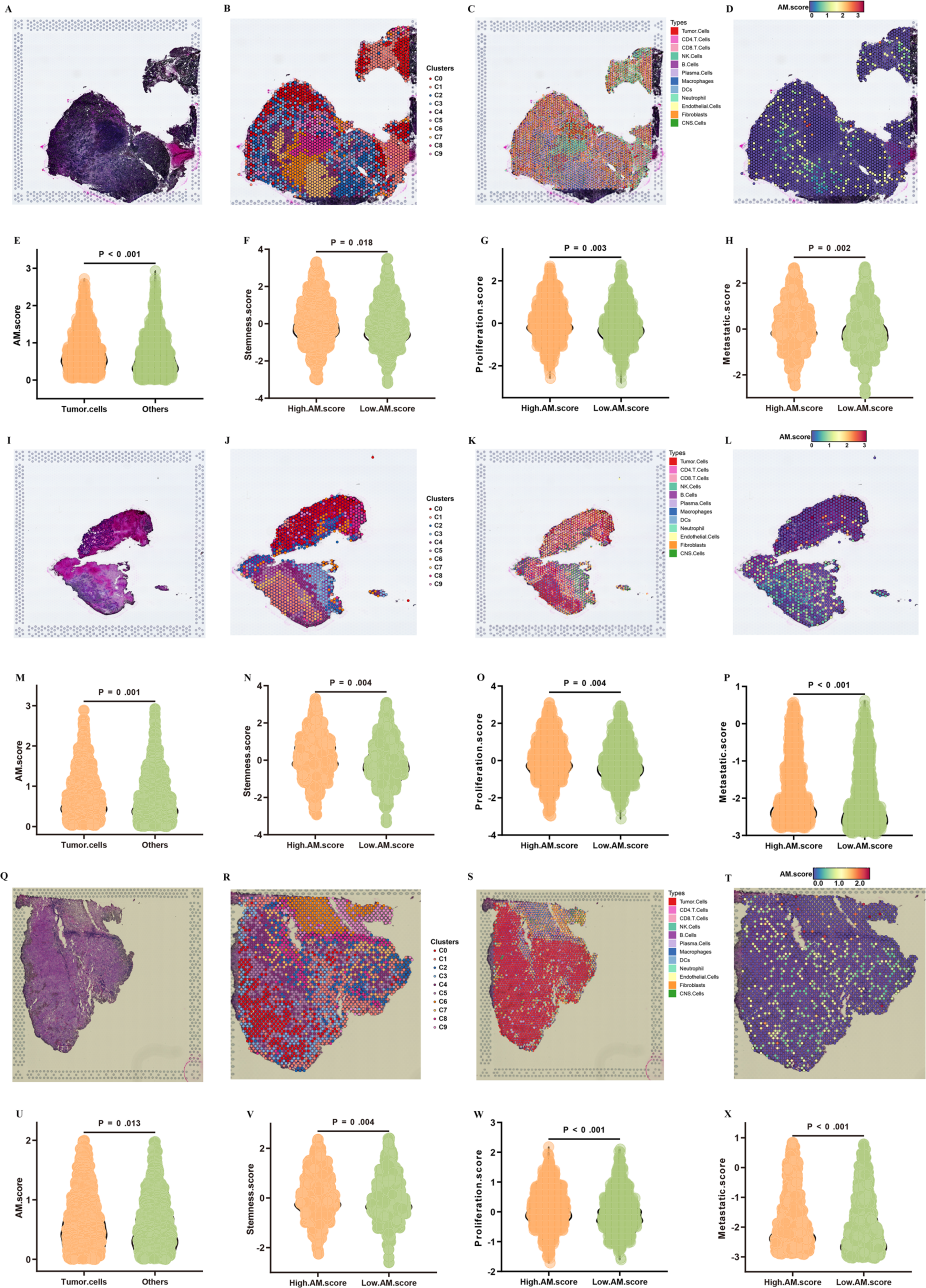
ST analysis of AM.score. Representative H&E staining of tissue sections of GSM5420749 **(A)**, GSM5420751 **(I)**, and GSM5420754 **(Q)**. Cell clusters were identified by performing UMAP of GSM5420749 **(B)**, GSM5420751 **(J)**, and GSM5420754 **(R)**. Cell types were identified by performing UMAP of GSM5420749 **(C)**, GSM5420751 **(K)**, and GSM5420754 **(S)**. The relative abundance of AM.score in cell types of GSM5420749 **(D)**, GSM5420751 **(L)**, and GSM5420754 **(T)**. Illustrating the differences in AM.score between the tumor cells and other cells in GSM5420749 **(E)**, GSM5420751 **(M)**, and GSM5420754 **(U)**. Violin plots displaying the expression scores of stemness in GSM5420749 **(F)**, GSM5420751 **(N)**, and GSM5420754 **(V)**, proliferation in GSM5420749 **(G)**, GSM5420751 **(O)**, and GSM5420754 **(W)**, and metastatic in GSM5420749 **(H)**, GSM5420751 **(P)**, and GSM5420754 **(X)** in tumor cells from the high AM.score and low AM.score groups.

Then, GSE179572 dataset includes specific samples identified as GSM5420749, GSM5420751, and GSM5420754, which were instrumental in exploring both the spatial distribution and the distinct expression characteristics of AM.score. By leveraging these samples, we aimed to uncover critical insights into how AM.score is represented across different contexts. The ST data were facilitated by thorough microscopic examination of H&E-stained sections (**Figures 7A, I, Q**). Following this initial phase, the gene expression data underwent normalization, then, PCA was implemented to identify genes characterized by variable expression. This analytical approach unveiled a total of 9 clusters in the GSM5420749, GSM5420749, GSM5420751, and GSM5420754 (**Figures 7B, J, R**). In the subsequent analysis, we annotated them into 12 cell types (**Figures 7C, K, S**). Our spatial transcriptomic analysis uncovered distinctive localization patterns of AM.score profiles within the tissues, highlighting the unique spatial organization of different cell populations (**Figures 7D, L, T**). Additionally, the findings indicate that tumor cells exhibiting higher AM.scores demonstrate a markedly increased expression of various markers that are linked to stemness, proliferation, and metastasis (**Figures 7E-H, M-P, U-X**).

### 3.8 ZDHHC18 overexpression associates with adverse prognosis in LUAD

**Figure 8:**
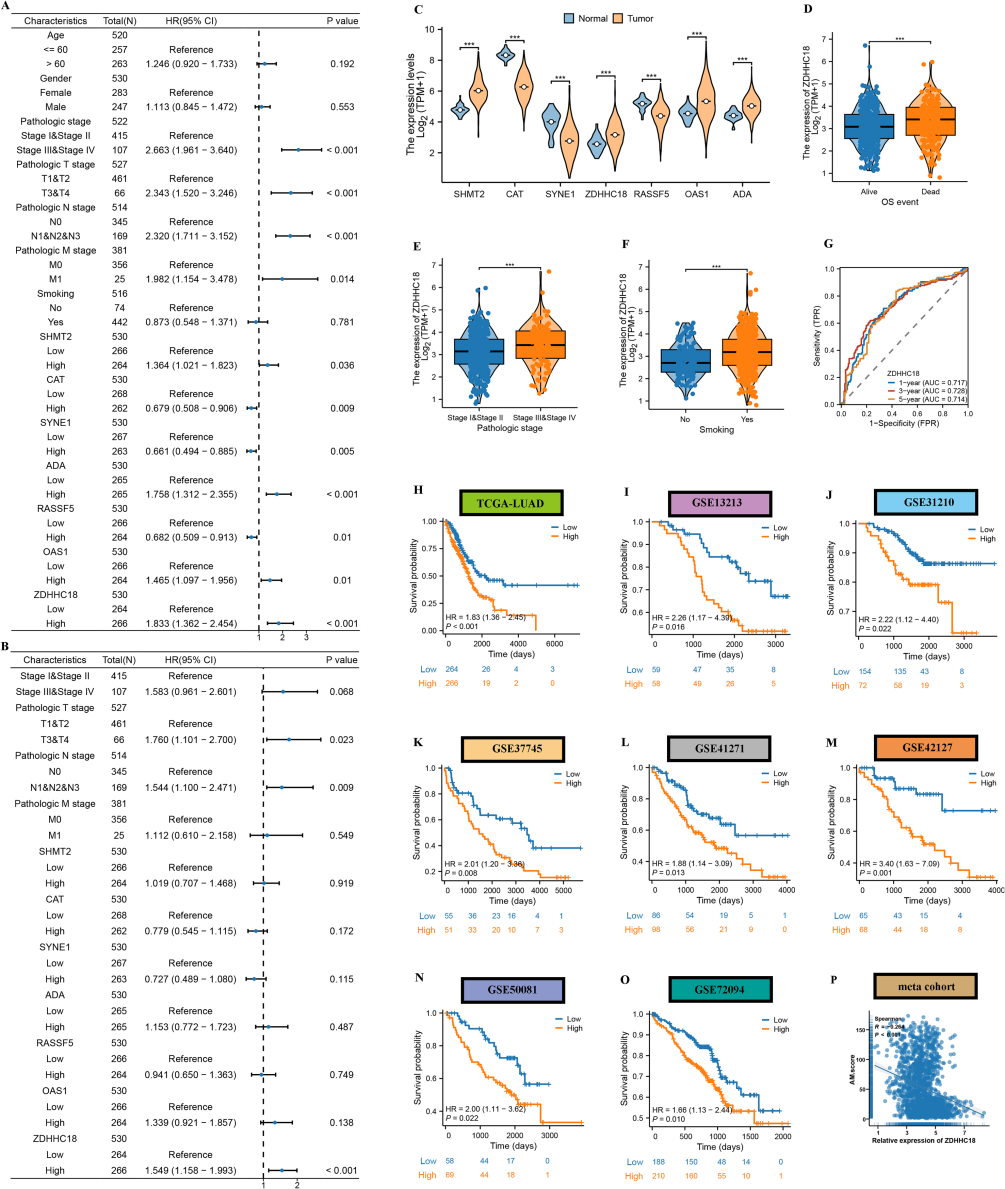
ZDHHC18 overexpression associates with adverse prognosis in LUAD. Univariate **(A)** and multivariate Cox regression **(B)** analyses of 7 genes and clinicopathological characteristics. **(C)** Analysis of differences of 7 genes between tumor and normal tissues. **(D-F)** Analysis of differences in ZDHHC18 among clinicopathological characteristics. **(G)** Time-dependent ROC curves of 1-year, 3-year, and 5-year OS of ZDHHC18. **(H-P)** Survival analysis of high and low ZDHHC18 groups.

Among the 20 genes, we identified seven genes (SHMT2, CAT, SYNE1, ZDHHC18, RASSF5, OAS1, and ADA) that exhibited differential expression between LUAD and normal samples while also serving as prognostic genes for LUAD (**Supplementary Figures 6A-V**). Subsequently, multivariate Cox regression analyses indicates that only ZDHHC18 maintains its considerable significance even when accounting for additional clinical parameters that are known to impact patient outcomes (**Figures 8A-C**). The findings of the study also revealed that patients categorized as being in a high stage of their condition, along with those who are smokers, demonstrated elevated levels of ZDHHC18 expression (**Figures 8D-F**). The predictive ability of ZDHHC18 for patient survival was also assessed using ROC curves, with AUC values 0.717, 0.728, 0.714 at one-, three-, and five-year intervals in TCGA-LUAD dataset, suggesting robust predictive capability (**Figure 8G**). Additional analysis of survival results revealed that individuals categorized under high ZDHHC18 exhibited notably worse survival when compared to those identified as belonging to low ZDHHC18 in the TCGA-LUAD, GSE41271, GSE37745, GSE42127, GSE50081, GSE72094, GSE13213, and GSE31210 datasets (**Figures 8H-O**). Moreover, the cohort exhibiting a high ZDHHC18 demonstrated a marked decrease in levels of immune cell infiltration in comparison to their counterparts with a low ZDHHC18 (**Supplementary Figures 7A-C**). The analysis indicated a significant negative relationship between the expression of ZDHHC18 and the AM.score (r=-0.264) (**Figure 8P**), highlighting ZDHHC18 as a key element within the AM.score framework.

### 3.9 ZDHHC18 knockdown inhibits LUAD malignant phenotypes

**Figure 9:**
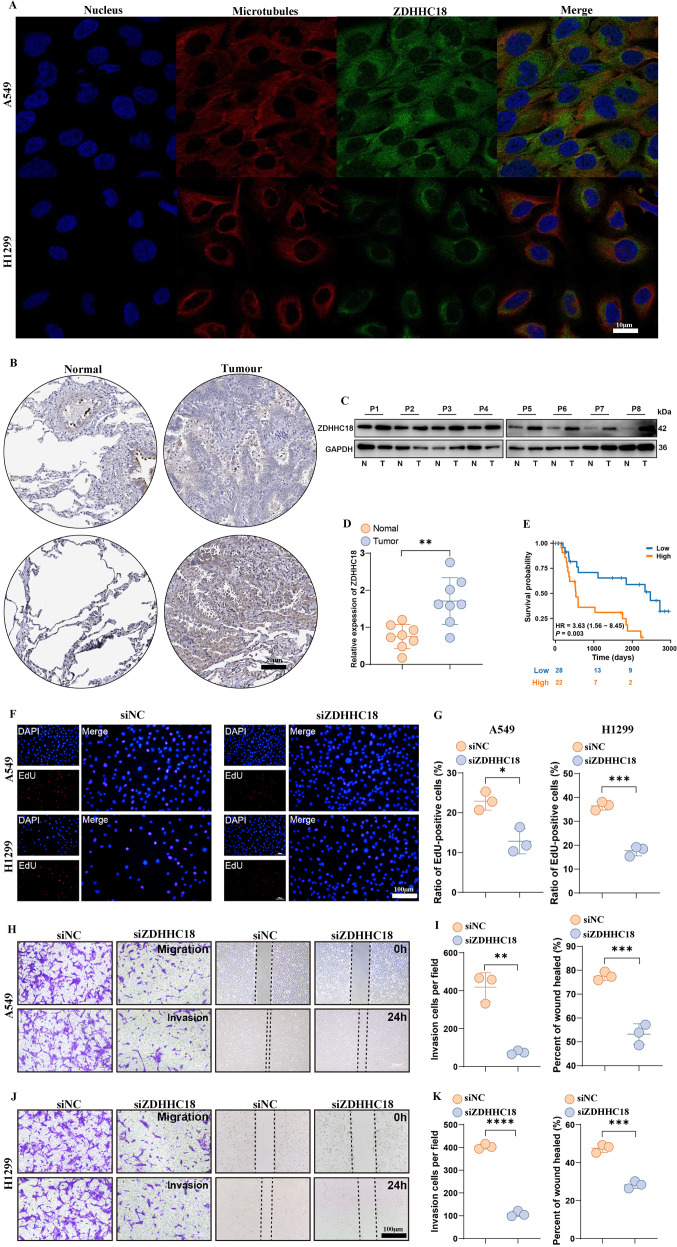
ZDHHC18 knockdown inhibits LUAD malignant phenotypes. **(A)** Fluorescent display of ZDHHC18 localization within cells. **(B)** Representative immunohistochemistry images of ZDHHC18 expression in normal tissues, LUAD tissue. ZDHHC18 protein levels **(C)** and mRNA **(D)** are shown for the LUAD and paired adjacent normal tissue. **(E)** Kaplan–Meier analysis of overall survival based on ZDHHC18 levels in 50 cases of LUAD patients. **(F, G)** EdU assay showing reduced growth in sh-ZDHHC18. Transwell migration and wound healing assays, demonstrating decreased migration and invasion ability in sh-ZDHHC18 A549 cells **(H, I)** and H1299 cells **(J, K)**. Data are presented as mean ± SD of three independent experiments. *p* < 0.01, **p < 0.001 indicate statistical significance compared to the siNC group.

IF experiments revealed ZDHHC18 was predominantly localized in the cytoplasm in tumor cells (**Figure 9A**). These cellular structures function as the principal locations for the process of intracellular protein palmitoylation, a post-translational modification that attaches palmitic acid to specific proteins. Validation studies conducted on clinical specimens revealed a reliable pattern of ZDHHC18 protein overexpression in tumor tissues when compared to adjacent normal tissues (**Figures 9B-D**). Additional analysis of survival results revealed that individuals categorized under high ZDHHC18 exhibited notably worse survival when compared to those identified as belonging to ZDHHC18 in house cohort (**Figure 9E**). Subsequently, we transfected H1299 and A549 cells with interfering fragments targeting ZDHHC18 and vector control plasmids, respectively. The results of the cell-scratch, and Trans well assays, show that the proliferative (**Figure 9F, G**), migratory (**Figures 9H, I**), and invasive (**Figures 9J, K**) abilities of the si-ZDHHC18 group were significantly decreased.

### 3.10 ZDHHC18 in C57BL/6 models

**Figure 10:**
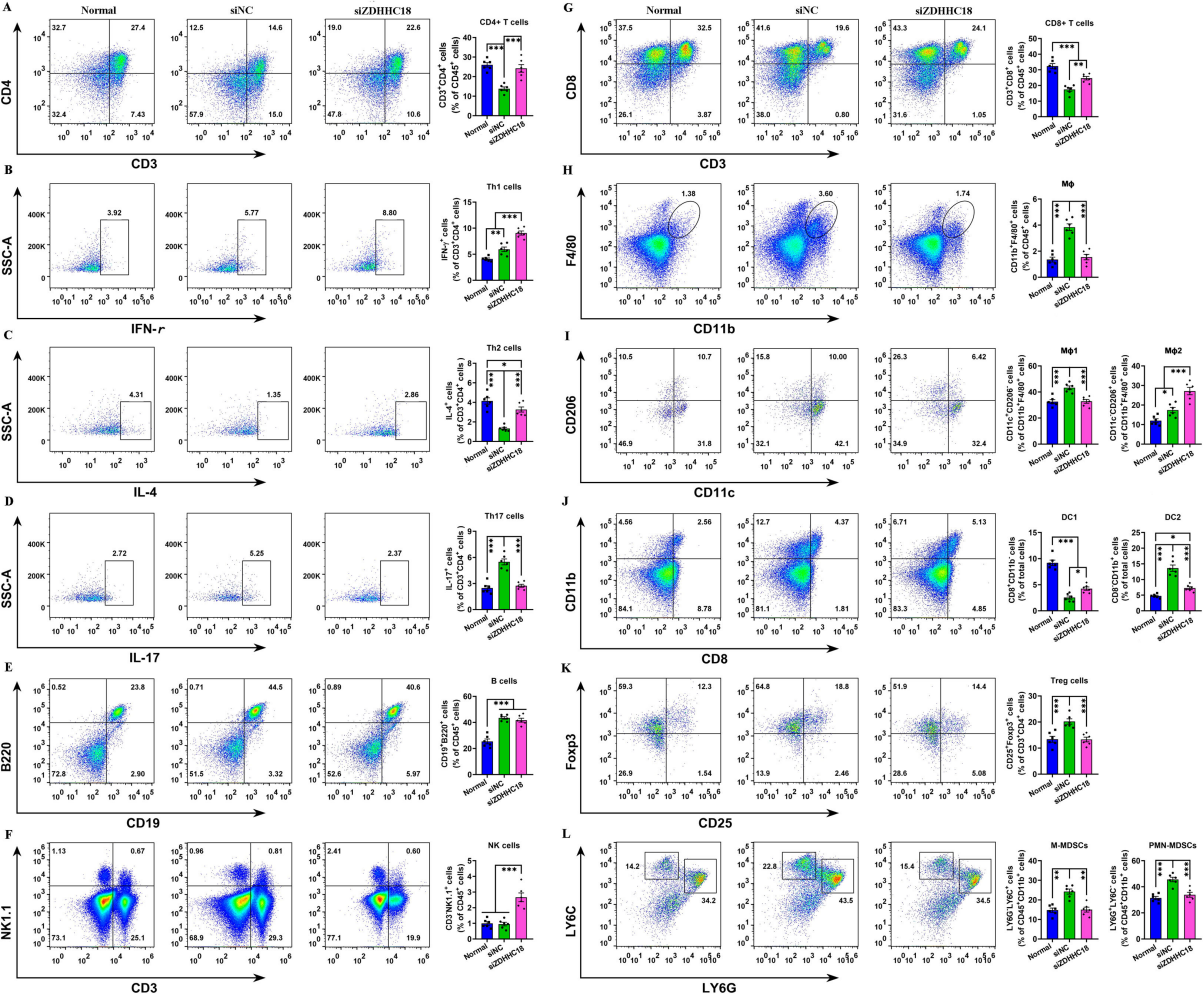
ZDHHC18’s role in immune regulation. FCM analysis of immune cell populations across 3 groups. **(A–D)** Representative FCM plots and statistical analysis of CD4 T cells (CD3+ CD4+ gated on CD45+), Th1 cells (IFN-γ+ gated on CD3+ CD4+ CD45+), Th2 cells (IL-4+ gated on CD3+ CD4+ CD45+), and Th17 cells (IL-17+ gated on CD3+ CD4+ CD45+). **(E)** FCM plots and quantification of B cells (CD19+ B220+), gated on CD3- CD4- CD45+ cells. **(F)** FCM plots and statistical analysis of NK cells (CD3- NK1.1+), gated on CD45+ cells. **(G)** FCM plots and quantification of CD8+ T cells (CD3+ CD8+), gated on CD45+ cells. **(H, I)** FCM plots and quantification of Mφ (CD11b+ F4/80+ gated on CD45+), Mφ1 (CD11c+ CD206- gated on CD11b+ F4/80+ CD45+) and Mφ2 (CD11c- CD206+ gated on CD11b+ F4/80+ CD45+). **(J)** FCM plots and statistical analysis of DC1 (CD8+ CD11b-) and DC2 (CD8- CD11b+), gated on CD45+ cells. **(K)** FCM plots and quantification of Treg cells (CD25+ Foxp3+), gated on CD3+ CD4+ CD45+ cells. **(L)** FCM plots and statistical analysis of M-MDSCs (LY6G- LY6C+) and PMN-MDSCs (LY6G+ LY6C-), gated on CD45+ CD11b+ cells. *p* < 0.01, **p < 0.001 indicate statistical significance compared to the siNC group.

To better understand the therapeutic efficacy and immunomodulatory properties of ZDHHC18, a study was conducted using C57BL/6 mice, which were randomly divided into three distinct experimental groups: normal mice, siNC, and siZDHHC18. In the context of immune alterations observed in the siZDHHC18 group as compared to the siNC group, it became evident that the manipulation of siZDHHC18 had a profound impact on the host’s anti-cancer immunity. Specifically, there was a noteworthy increase in the proportion of immune cells exhibiting positive activity within the LUAD microenvironment. This indicates that the immune response is being effectively bolstered in the presence of siZDHHC18. Conversely, when examining immunosuppressive cells, a significant reduction in their proportions was noted following the administration of siZDHHC18 (**Figures 10A-L**). This decline in immunosuppressive cell populations suggests that intervention with siZDHHC18 not only enhances the activation of anti-cancer immune cells but also diminishes the factors that typically hinder immune responses against tumors. Overall, these findings highlight the potential of siZDHHC18 in strengthening host immunity while concurrently reducing immunosuppression in the context of LUAD.

## 4 Discussion

Metastasis is the primary cause of death in the vast majority of cancer patients, with its biological processes involving multiple aspects such as tumor cell phenotypic plasticity, metabolism, epigenetic regulation, and tumor microenvironment (TME) (34). Recent studies indicate that abnormal expression and function of acylation modification proteins (AMPs) are associated with cancer metastasis, with many AMPs involved in post-transcriptional regulation exhibiting dysregulation during tumor metastasis. During cancer metastasis, AMPs not only regulate RNA localization, metabolism, and function at the post-transcriptional level but also influence DNA and protein functions (35, 36). Consequently, they can broadly impact various biological processes associated with metastasis. Therefore, in-depth investigation into therapeutic strategies targeting AMPs holds significant promise for the treatment of tumor metastasis.

Initially, enrichment analysis indicated significant enrichment of DEGs and WGCNA between the various subtypes studied. Notably, these DEGs and modular genes showed a strong predominance in pathways related to DNA replication and immune pathways associated with LUAD. These processes are critically intertwined with the mechanisms underlying the pathogenesis of LUAD, suggesting that the observed genetic differences could play a pivotal role in the onset and progression of this condition. Targeted therapies focusing on DNA replication have shown definite efficacy in improving progression of LUAD, and are expected to become one of the effective strategies for the treatment of LUAD.

Then, our extensive research on AM has underscored the crucial role these modifications play in progression of LUAD. By analyzing data from 1,720 LUAD across eight international studies, we have successfully created a sophisticated computational model named AM.score. This model is meticulously designed to capture the prognostic relevance of 20 significant AM linked to the disease, thereby enhancing our understanding of their impact on LUAD. Moreover, clinical utility of the AM.score model extends well beyond lung adenocarcinoma alone. It has been rigorously validated in 9 separate immunotherapy cohorts, encompassing a wide array of cancer types. Notably, AM.score has demonstrated a remarkable capability for prognostic accuracy, outperforming 7 existing signatures related to LUAD. The KEGG pathway enrichment analysis results of this study indicate that the DEGs between high/low AM.score are primarily enriched in signaling pathways such as the mTOR signaling pathway. As a central regulator of metabolic and immune processes, mTOR is closely linked to acylation modification and tumor microenvironment regulation (37–41). This impressive accuracy firmly establishes AM.score as an invaluable tool for customizing immunotherapy approaches, ultimately aiming to enhance treatment efficacy and improve patient outcomes in a variety of cancer settings.

The TME is crucial in the advancement and progression of different types of cancer. We propose that the precise prognostic predictions made by the AM.score in patients with LUAD could be associated with the TME. By conducting a thorough analysis of various immune cells present within the microenvironment, we found that a high AM.score is significantly associated with increased levels of immune cell infiltration. This observation stands in stark contrast to the characteristics exhibited by those with a low AM.score, highlighting the potential implications of AM.score as a predictive marker for immune response. The pronounced infiltration of immune cells in specimens with elevated AM.scores suggests an enriched immune landscape, which could play a critical role in understanding the dynamics of immune activity in different physiological and pathological contexts. Reports indicate that CAFs promote tumor progression through multiple mechanisms, including enhancing fibrosis within the malignant cells and the stromal tumor microenvironment (42). Dendritic cells are crucial in the immune system as they function as antigen-presenting cells. Their primary role involves initiating and regulating immune responses, effectively acting as a vital link between the innate and adaptive immune systems. This connection is essential for coordinating the body’s defense mechanisms against pathogens, as dendritic cells capture and process antigens, presenting them to T cells and thereby facilitating a robust immune response (43, 44). In addition to dendritic cells, endothelial cells play important roles in regulating both innate and adaptive immune responses. These cells are not merely structural components of blood vessels; they actively participate in the immune response by performing several key functions. Endothelial cells are involved in the secretion of cytokines, which are signaling molecules that help modulate the immune response. Furthermore, they contribute to antigen presentation and possess anti-inflammatory properties, which help to regulate inflammation and maintain homeostasis within the immune system (45, 46). Together, dendritic cells and endothelial cells form a sophisticated network that enhances the body’s ability to respond to immunological challenges effectively.

Clinically, immune checkpoint inhibitors (ICIs) have shown strong and lasting antitumor effects in individuals with LUAD. Nevertheless, the response rate to therapy using immune checkpoint inhibitors is still quite limited, with only a portion of LUAD patients experiencing any benefits (47, 48). Up to the present, numerous biomarkers have been identified that may serve as indicators for predicting the effectiveness of ICIs therapy (49, 50). Despite the introduction of these promising biomarkers, the results regarding their predictive capacity have not met expectations. As a result, the search for reliable markers that can accurately forecast therapeutic outcomes continues to be a significant challenge in the field. We observed that AM.score values were significantly higher in the group receiving either ICIs, compared to other groups. This suggests our model may assist in selecting patients prior to treatment with immune checkpoint inhibitors.

In further exploring the underlying mechanisms of AM.score, we observed ZDHHC18 has been recognized as an essential molecular element in the framework. Protein palmitoylation is a common form of acylation modification that plays a crucial regulatory role in protein transport, localization, and functional activation, and is facilitated by a specific group of enzymes known as zinc finger DHHC (ZDHHC) palmitoyl transferases (51). In the context of protein transport, DHHC2 can interact with proteins on the cell membrane to form a dynamic circular pathway on the cell membrane, thereby mediating protein palmitoylation modification and transport (52). Concurrently, the cysteine (Cys)-terminal domain of DHHC2 regulates its membrane localization and associated enzymatic activity, thereby exerting regulatory functions over protein palmitoylation and transport (8a). In signal transduction, specific sites on the membrane protein SNAP25 within neurons and endocrine cells can be catalyzed by DHHC proteins to undergo palmitoylation. This influences its interactions with other proteins and signal transduction pathways, participating in the regulation of neurotransmitter and insulin secretion processes (53). In the context of immune responses, ZDHHC18 is rapidly activated following T cell receptor (TCR) activation and modulates the activity of multiple signaling molecules, including LCK, ZAP70, LAT, and RasGRP1, via palmitoylation. Furthermore, the absence of ZDHHC18 leads to T cell dysfunction and the development of autoimmune diseases (54). In the clinical samples of this study, ZDHHC18 expression was higher in LUAD tissues than in adjacent normal tissues. By stably establishing a cell line with low ZDHHC18 expression through siRNA-mediated suppression, we conducted *in vitro* experiments including Western blot analysis, PCR assays, and cell cloning assays. These revealed a marked reduction in cellular proliferation following siRNA-mediated ZDHHC18 suppression. *In vivo* experiments involving subcutaneous tumor formation in mice revealed knockout ZDHHC18 simultaneously enhance strong immune responses that hinder LUAD progression.

Although the therapeutic potential of ZDHHC18 appears promising, it is important to acknowledge some methodological limitations that need to be addressed. One significant concern is that the analysis relied on a limited number of LUAD, resulting in relatively small sample sizes, especially among patients diagnosed with LUAD. To enhance the robustness and generalizability of future research, it is essential that studies engage larger, independent cohorts and take into account the presence of comorbid conditions. Further comprehensive research is necessary to elucidate the molecular mechanisms that connect ZDHHC18 to the growth of LUAD and the phenomenon of immunosuppression. Currently, our understanding remains incomplete, and additional studies are essential to uncover how ZDHHC18 influences these critical aspects of LUAD progression and immune response. Such investigations could provide valuable insights into the role of ZDHHC18 in cancer biology and its potential implications for therapeutic strategies.

## 5 Conclusions

In conclusion, a comprehensive analysis was performed to uncover acylation modification related to ZDHHC18 and LUAD. This study offers significant genetic insights that highlight the therapeutic potential of targeting ZDHHC18 in LUAD. Through a comprehensive study of ZDHHC18 in LUAD, we improve our insight into the core mechanisms underlying the condition. This exploration can shed light on the complex biological processes involved in LUAD and suggesting innovative and promising strategies for effective treatment options, paving the way for more targeted therapeutic interventions in managing the disease.

## Data Availability

The original contributions presented in the study are included in the article/**Supplementary Material**. Further inquiries can be directed to the corresponding author.

## Supplementary material

Supplementary Table 1 The acylation modification related genes used in this study.

Supplementary Figure 1 Principal component analysis (PCA) of eight datasets before **(A)** and after **(B)** batch effect removal.

Supplementary Figure 2 **(A)** The Cluster Prediction Index and Gap Statistical Analysis of the multiomics clusters. **(B)** Consensus clustering matrix for two novel prognostic subtypes based on the 10 algorithms.

Supplementary Figure 3 **(A)** The sample similarity of each subgroup was assessed by calculating the Silhoutte score. **(B)** Identification of co-expression gene modules.

Supplementary Figure 4 **(A-H)** Time-dependent ROC curves of 1-year, 2-year, and 3-year OS for AM.score.

Supplementary Figure 5 **(A)** The immune landscape between the high AM.score and low AM.score groups. **(B)** The immune modulators between the high AM.score and low AM.score groups.

Supplementary Figure 6 **(A-B)** Analysis of differences of 20 genes between tumor and normal tissues. **(C-V)** Survival analysis of high and low of 20 genes.

Supplementary Figure 7 **(A-C)** The immune landscape between the high ZDHHC18 and low ZDHHC18 groups.
