# Supplementary figures and images for: Acylation modification mediated post-translational modifications learning signature reveals ZDHHC18 promotes progression of lung adenocarcinoma by attenuating immunocyte activation
Source: Front Immunol. 2026 Apr 7;17:1802631. doi: 10.3389/fimmu.2026.1802631 (PMC13095696; doi:10.3389/fimmu.2026.1802631)

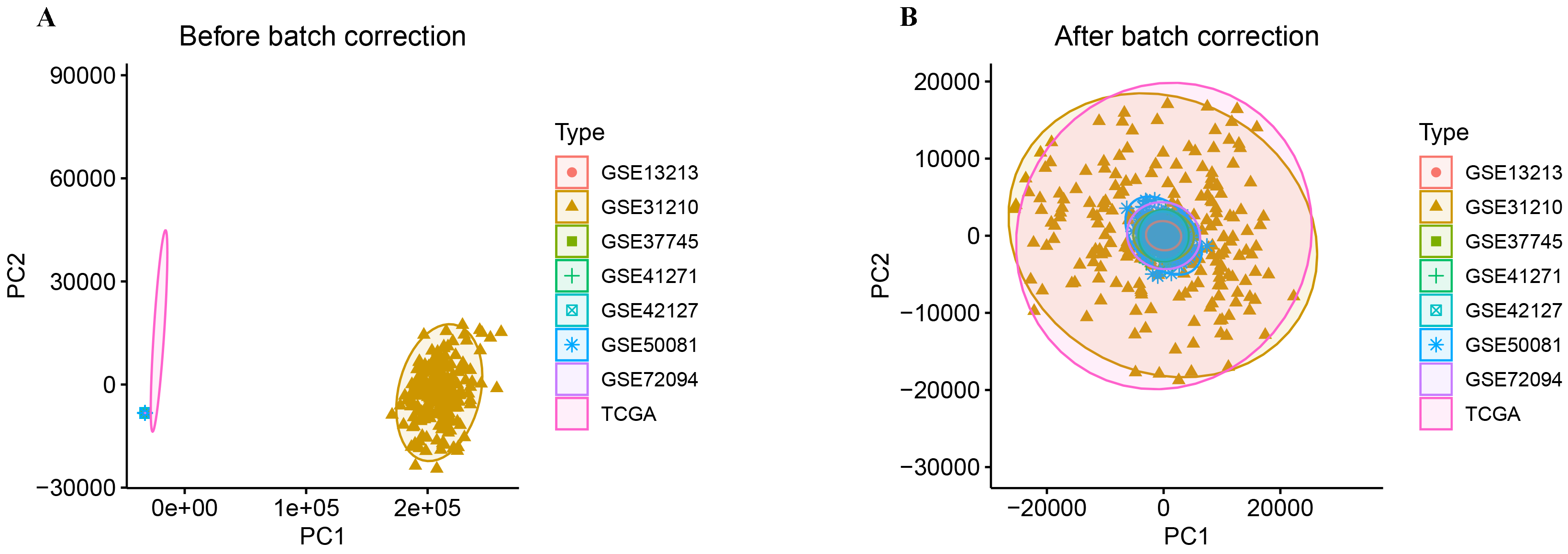

Supplement: Supplementary Figure 1 — Principal component analysis (PCA) of eight datasets before (A) and after (B) batch effect removal. [file Image1.tif]

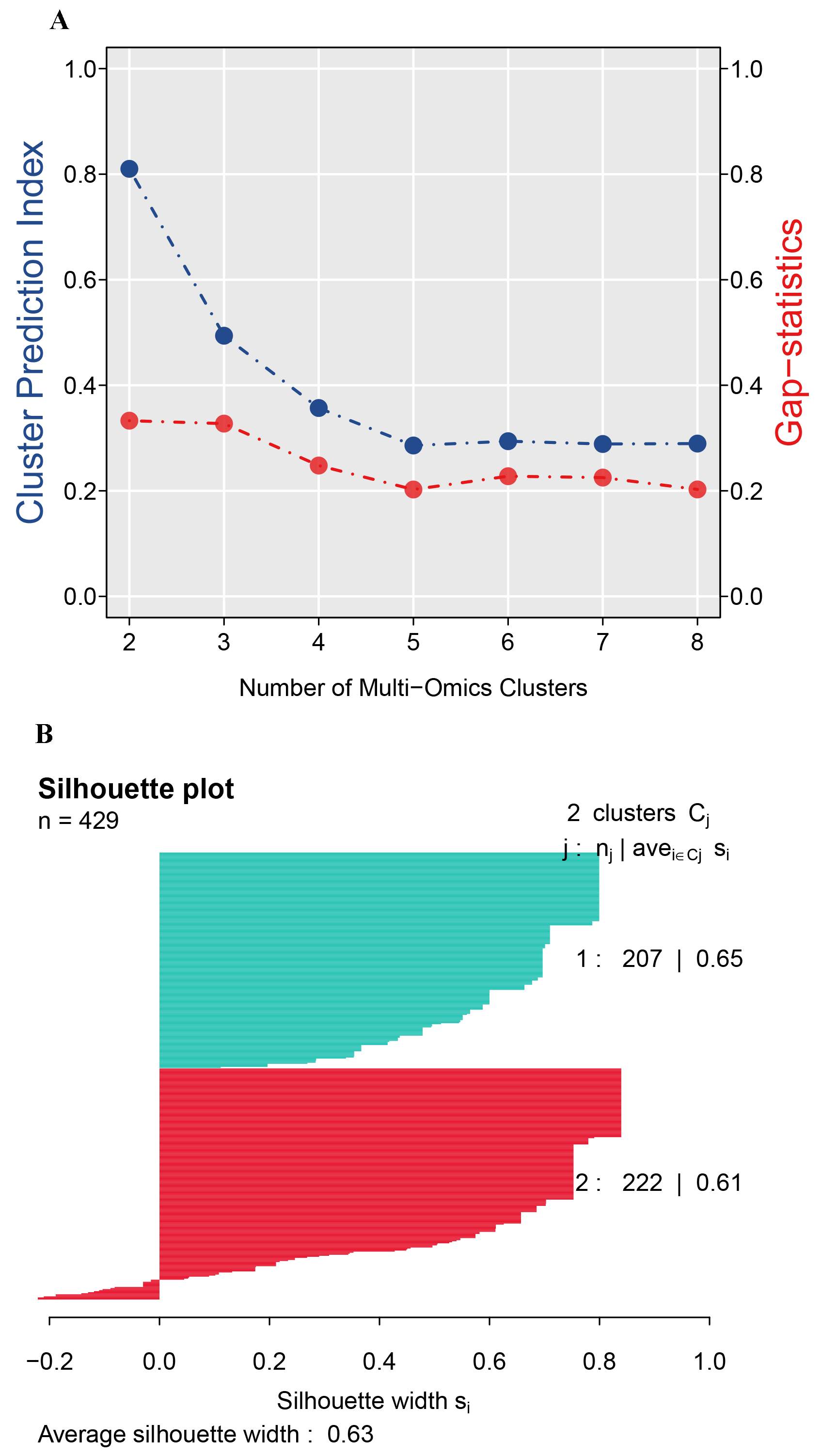

Supplement: Supplementary Figure 2 — (A) The Cluster Prediction Index and Gap Statistical Analysis of the multiomics clusters. (B) Consensus clustering matrix for two novel prognostic subtypes based on the 10 algorithms. [file Image2.tif]

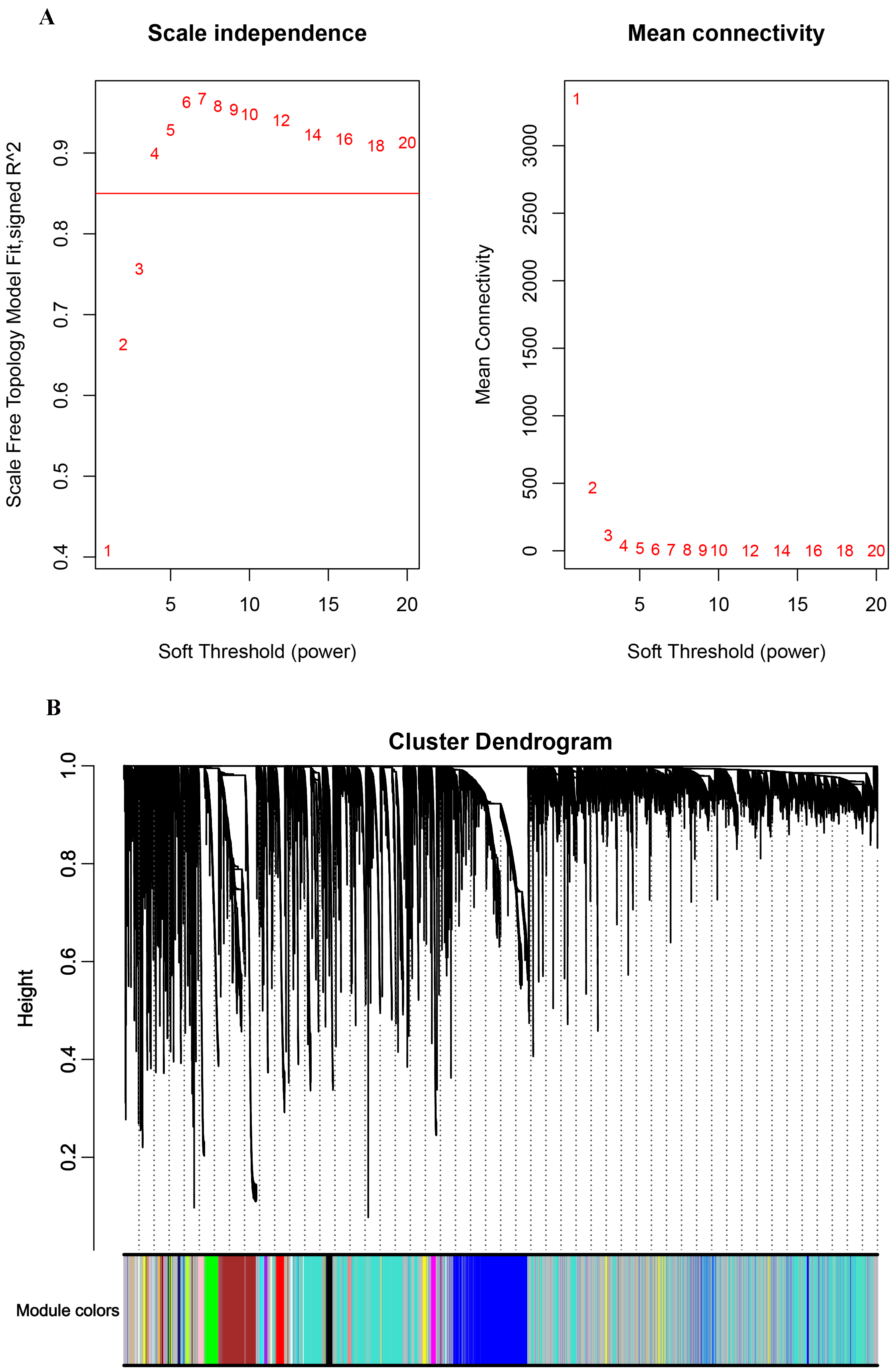

Supplement: Supplementary Figure 3 — (A) The sample similarity of each subgroup was assessed by calculating the Silhoutte score. (B) Identification of co-expression gene modules. [file Image3.tif]

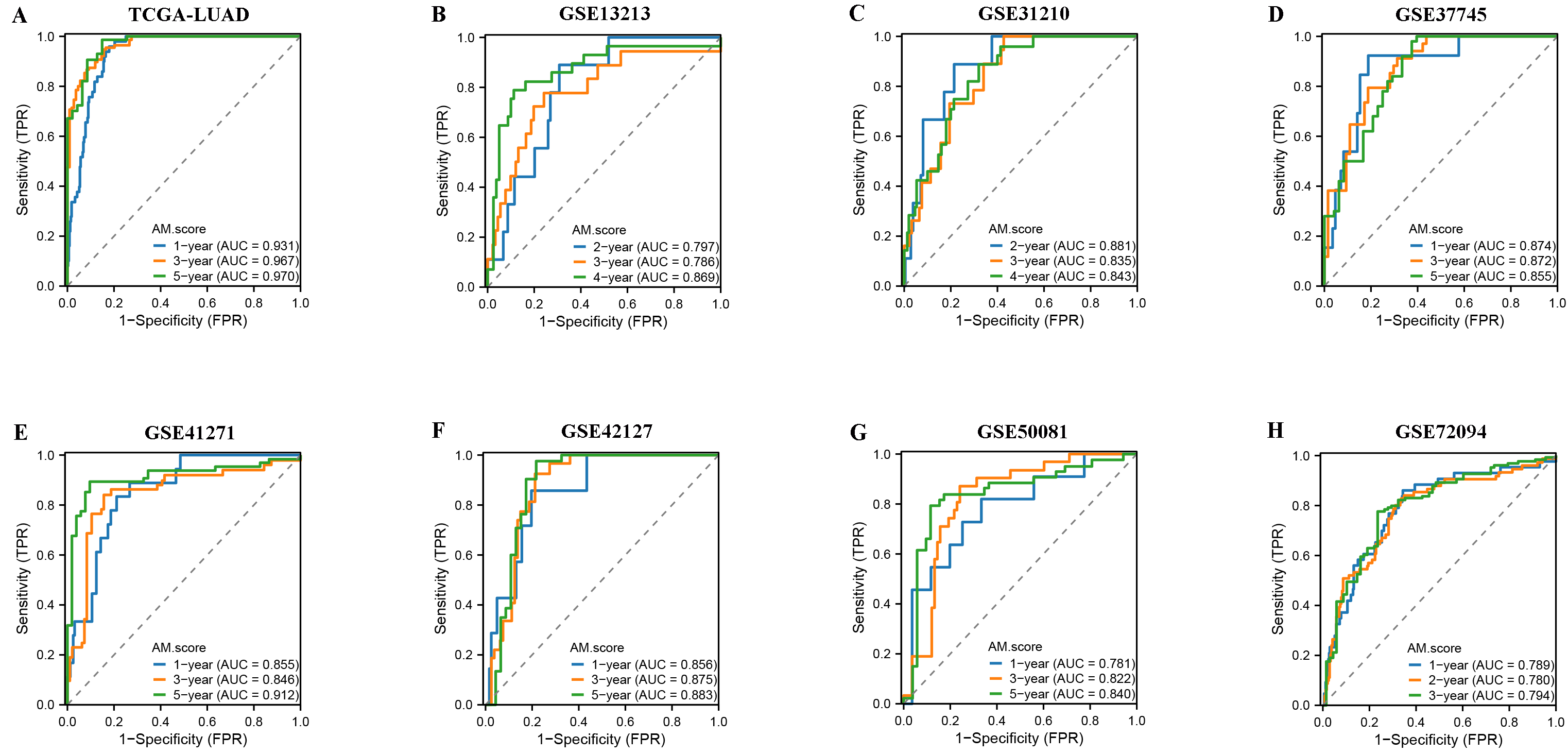

Supplement: Supplementary Figure 4 — (A-H) Time-dependent ROC curves of 1-year, 2-year, and 3-year OS for AM.score. [file Image4.tif]

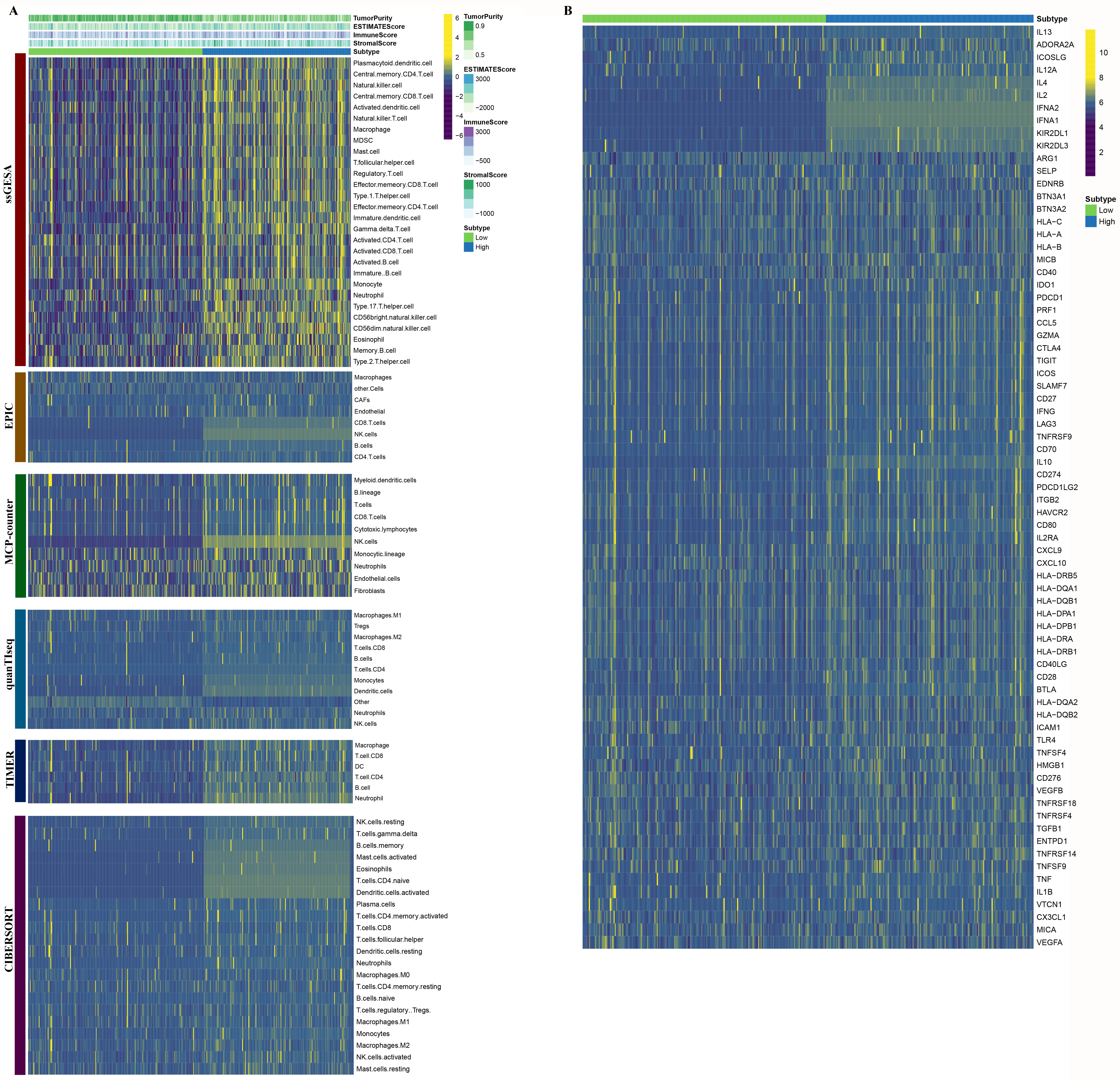

Supplement: Supplementary Figure 5 — (A) The immune landscape between the high AM.score and low AM.score groups. (B) The immune modulators between the high AM.score and low AM.score groups. [file Image5.tif]

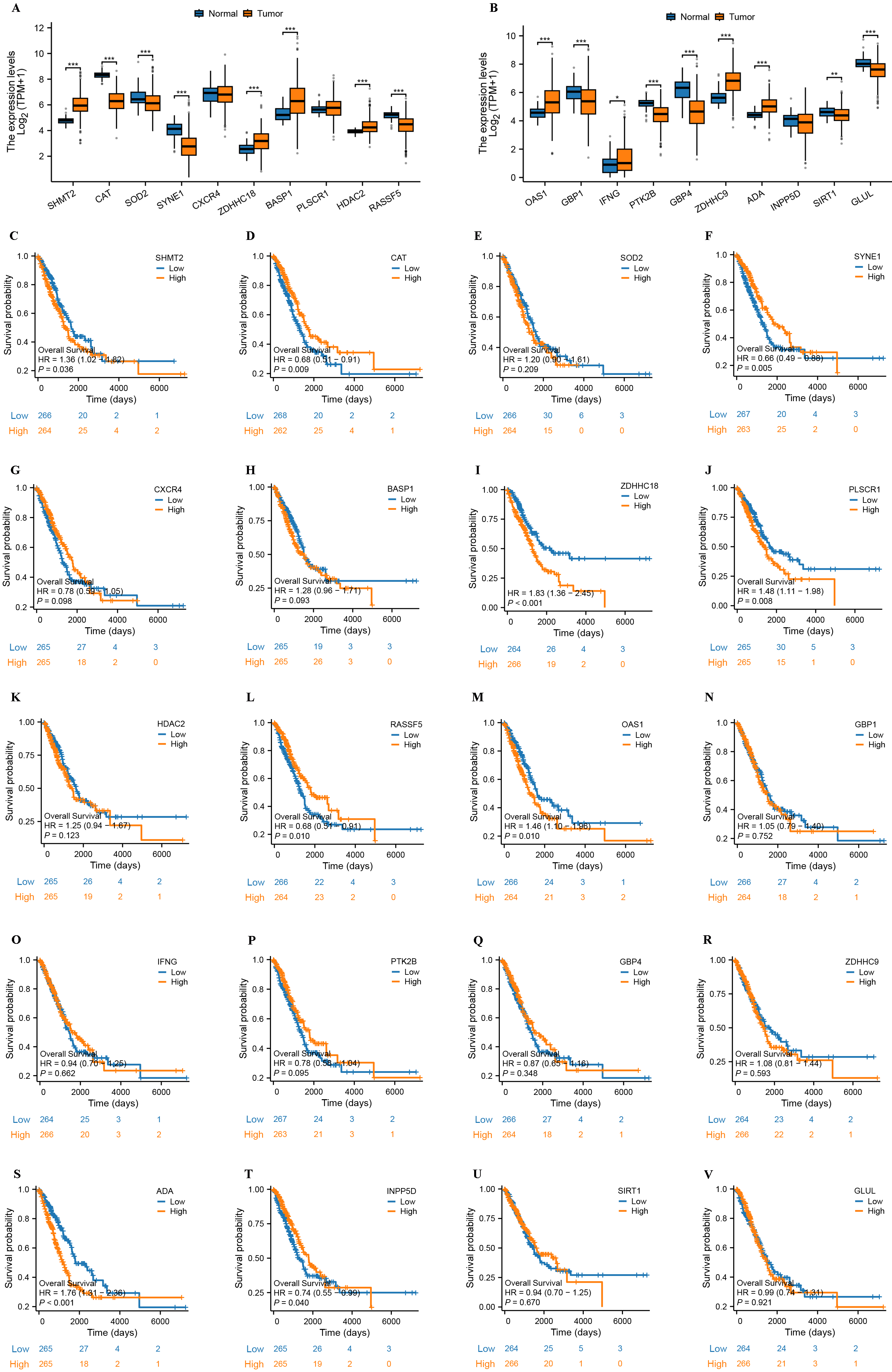

Supplement: Supplementary Figure 6 — (A-B) Analysis of differences of 20 genes between tumor and normal tissues. (C-V) Survival analysis of high and low of 20 genes. [file Image6.tif]

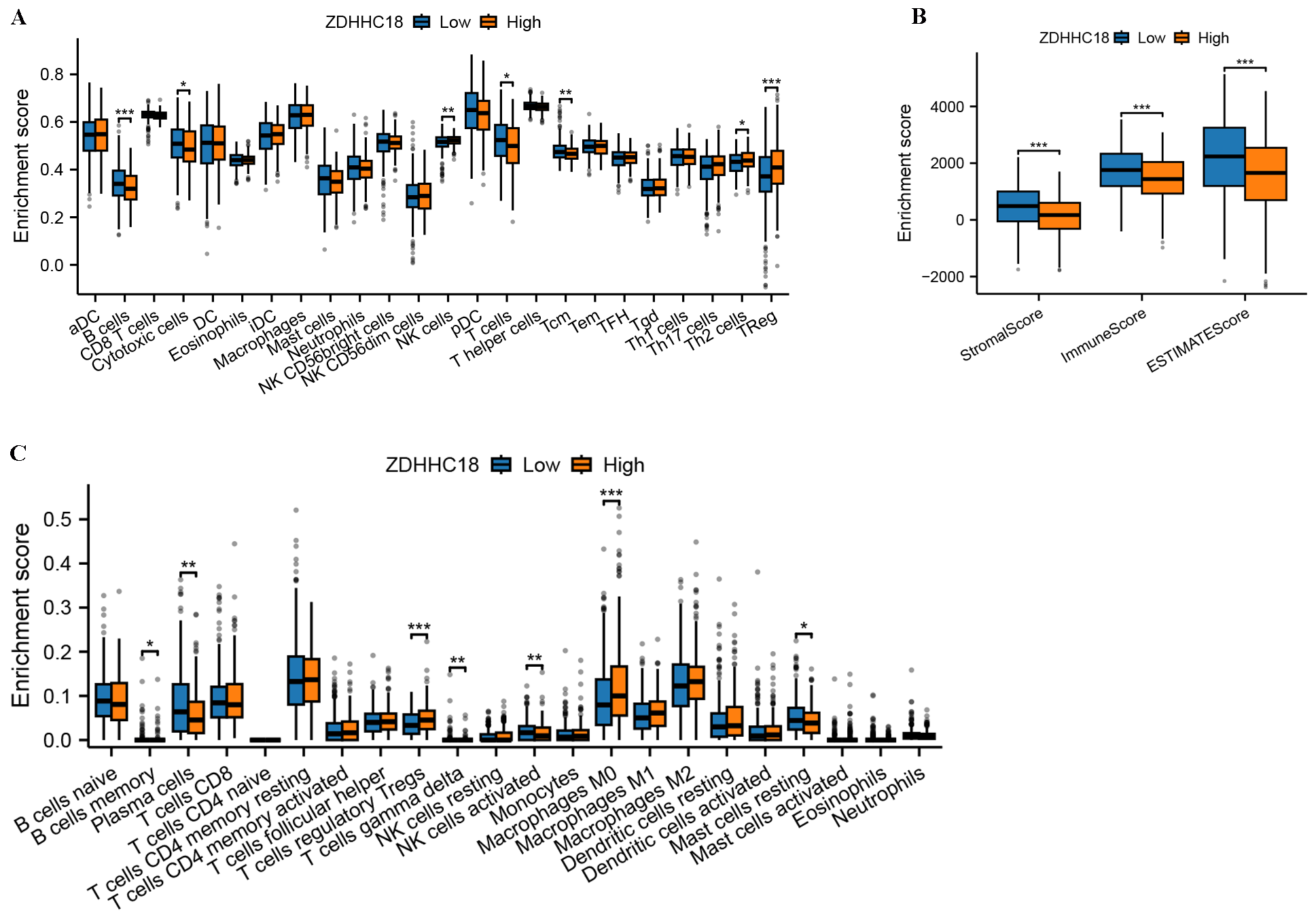

Supplement: Supplementary Figure 7 — (A-C) The immune landscape between the high ZDHHC18 and low ZDHHC18 groups. [file Image7.tif]
